# Analysis of COVID-19 vaccine adverse event using language model and unsupervised machine learning

**DOI:** 10.1371/journal.pone.0282119

**Published:** 2023-02-21

**Authors:** Saeyeon Cheon, Thanin Methiyothin, Insung Ahn

**Affiliations:** 1 Department of Data-Centric Problem Solving Research, Korea Institute of Science and Technology Information, Daejeon, Republic of Korea; 2 Center for Convergent Research of Emerging Virus Infection, Korea Research Institute of Chemical Technology, Daejeon, Republic of Korea; 3 Department of Applied AI, University of Science & Technology, Daejeon, Republic of Korea; Université Paris 13 UFR de Santé Médecine Biologie Humaine: Universite Sorbonne Paris Nord UFR de Sante Medecine Biologie Humaine, FRANCE

## Abstract

**Background:**

After the COVID-19 pandemic, the world has made efforts to recover from the chaotic situation. Vaccination is a way to help control infectious diseases, and many people have been vaccinated against COVID-19 by this point. However, an extremely small number of those who received the vaccine have experienced diverse side effects.

**Methods and findings:**

In this study, we examined people who experienced adverse events with the COVID-19 vaccine by gender, age, vaccine manufacturer, and dose of vaccinations by using the Vaccine Adverse Event Reporting System datasets. Then we used a language model to vectorize symptom words and reduced their dimensionality. We also clustered symptoms by using unsupervised machine learning and analyzed the characteristics of each symptom cluster. Lastly, to discover any association rules among adverse events, we used a data mining approach. The frequency of adverse events was higher for women than men, for Moderna than for Pfizer or Janssen, and for the first dose than for the second dose. However, we found that characteristics of vaccine adverse events, including gender, vaccine manufacturer, age, and underlying diseases were different for each symptom cluster, and that fatal cases were significantly related to a particular cluster (associated with hypoxia). Also, as a result of the association analysis, the {chills ↔ pyrexia} and {vaccination site pruritus ↔ vaccination site erythema} rules had the highest support value of 0.087 and 0.046, respectively.

**Conclusions:**

We aim to contribute accurate information on the adverse events of the COVID-19 vaccine to relieve public anxiety due to unconfirmed statements about vaccines.

## Introduction

A worldwide pandemic of the novel coronavirus spread rapidly starting in early 2020, and had a severe effect on millions of lives and the global economy, with varying degrees of severity and mortality rates, depending on factors such as comorbidities [1, 2]. Vaccinations are an effective way to end this pandemic [1], and multiple countries are actively vaccinating their citizens to eradicate the virus. According to the COVID-19 vaccination dataset, 43.7% of the global population had completed fully vaccination as of November 2021 [3]. This result means that many countries, as well as individuals, are preparing to return to normalcy.

Some of these vaccines were developed using transformative new technologies to exploit mRNA. However, the unavoidable, rare adverse events can cause concern among the general public. We hope to illuminate the statistics of adverse events in this study, using real data and objective methods.

**Fig 1:**
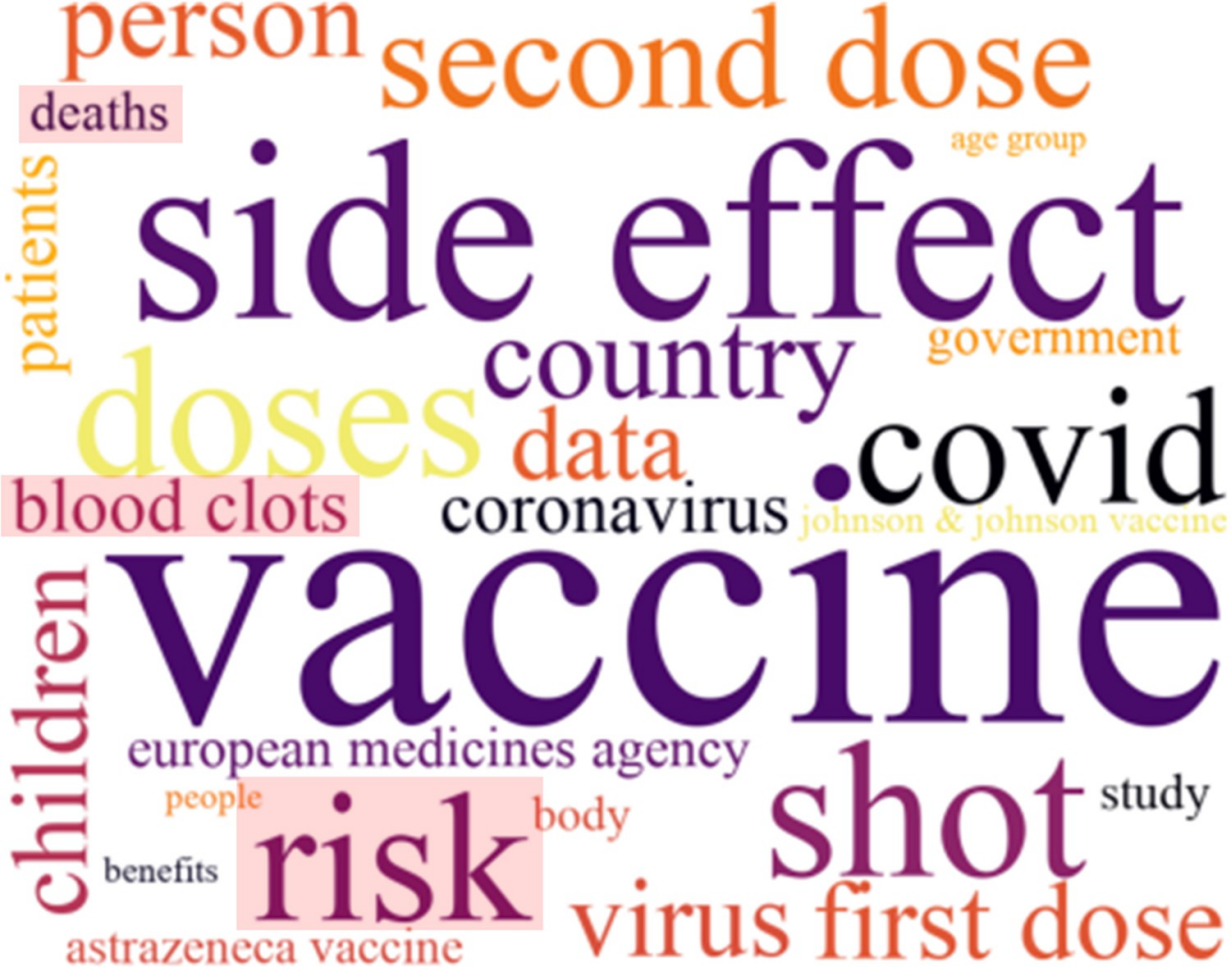
Word cloud of top 26 keywords regarding side effects of the COVID-19 vaccine from Meltwater. Keywords include negatively connoted words such as risk, blood clots, and deaths.

We used Meltwater (Media Monitoring and Social Listening Platform, www.meltwater.com) and Google Trends (trends.google.com/trends/) to investigate social media reactions to the side effects of the COVID-19 vaccine. Meltwater is a platform that provides news and various social network services (SNSs) data concerning Twitter, blogs, YouTube, and other social media platforms, and simple data analysis. Google Trends provides data regarding the search volume for various keywords searched using Google. On Meltwater, we set (“covid” and “vaccine” and “side effect” not “RT”) as a search query from November 2020 to October 2021 and received 291,960 total mentions, most frequently in April. As shown in Fig 1, the keyword analysis confirmed that some negative words such as risk, blood clots, and deaths were mentioned. Similarly, when we analyzed the result of the search word “covid vaccine side effect” on Google Trends over the same period, the highest interest was found in April 2021. Various symptoms showed as a result of related topics and queries. Furthermore, we confirmed that when “covid vaccine” is searched, “side effect” and “side effects covid vaccine” rank as the 7th most popular related topic and 12th most popular related query, respectively. The results of our analysis of news, SNS, and Google searches indicated significant interest in the side effects of the COVID-19 vaccine.

The side effects of the COVID-19 vaccine exhibit various symptoms, so clear information is difficult to obtain. Therefore, we applied data mining techniques to actual data about adverse events to analyze the symptoms and characteristics associated with adverse events to the vaccine. Other studies have pursued a similar goal of analyzing adverse events of COVID-19 vaccines [4, 5]. Some focused on four of the more common adverse reactions such as cerebral thrombosis, Guillain-Barré syndrome, myocarditis, and pericarditis [6, 7]. Moreover, other study was performed using data collected from the smartphone-based application [8] and evaluated the epidemiological of post-vaccine using adverse event data [9].

We used the Vaccine Adverse Event Reporting System (VAERS) data to analyze adverse event information on various vaccines [10–13], and applied data mining methods. Data mining is used in many fields to discover and extract meaningful information from big data [14]. In particular, association rule analysis is one of the data mining techniques to identify frequent patterns in transactions and to quantify their usefulness [15–17]. Recently, this technique has also been shown to be useful in the biomedical field and related studies [18–20].

We collected the total number of vaccinations in the United States from Our World in Data to analyze with the VAERS data [10]. As of August 31, 2021, the total number of vaccinations is 369,860,644 doses, including 210,054,512 Pfizer doses (57%); 145,447,491 Moderna doses (39%); and 14,358,641 Jansen doses (4%).

We analyzed adverse case statistics, emergency room visits, fatalities, and allergy data associated with medications or other products. The ratio of total adverse reactions was ranged from ~ 1/1200 (Pfizer) to ~ 1/500 (Janssen) and the mortality rate ranged from ~1/120,000 (Pfizer) to ~1/43,000 (Janssen) as presented in Table 1. Although the numbers of adverse cases are small, anyone who has been vaccinated can be concerned about the possibility of experiencing them. In other words, the actual fear index might be much greater than the incidence rate.

**Table 1:** The ratio of vaccinated with adverse cases, emergency room visits, deaths, and allergies.

	Pfizer	%	Moderna	%	Janssen	%
**Vaccinated**	210,054,512	-	145,447,491	-	14,358,641	-
**Adverse case**	165,061	0.0789	203,391	0.1398	27,991	0.1949
**ER**	26,427	0.0126	20,819	0.0143	4,558	0.0317
**Died**	1,736	0.0008	2,154	0.0015	329	0.0023
**Adverse with personal allergy record**	48,235	0.0232	64,270	0.0442	9,785	0.0681
**Adverse with personal allergy record and ER**	9,148	0.0044	8,442	0.0058	1,816	0.0126
**Adverse with personal allergy record and died**	421	0.0002	521	0.0004	93	0.0006

In this study, we analyzed adverse event symptoms of the COVID-19 vaccine in the VAERS dataset. Then we vectorized adverse event symptoms by using Word2Vec language model [21] and reduced the dimensionality of symptom vectors. Then we clustered the results into groups by using Density-based spatial clustering of applications with noise (DBSCAN) unsupervised machine learning model [22] and analyzed the characteristics of symptom clusters. We also identified association rules between symptoms by using the Apriori algorithm [16] and used the results to determine as to which symptoms may be comorbid.

The aim of this work is to enable prospective vaccination recipients to fully recognize by suggesting tendency of adverse events and comfortably decide on vaccination. Moreover, the results can help to relieve public anxiety of distrust and to refute false information about COVID-19 vaccines.

## Method

### Data sources

The VAERS dataset is a vaccine adverse event dataset provided by the CDC and the US FDA [10]. It is a national early warning system that is used to monitor the safe administration of licensed vaccines in the US. VAERS receives adverse event reports from healthcare providers, vaccine manufacturers, and the public through a standard form that includes partially narrative text input. The reports can be submitted by online, mail, or fax. Then medical terms are assigned by specialists using the Medical Dictionary for Regulatory Activities (MedDRA) schema [23]. However, it is especially notable that not all VAERS reports contain complete, precise, and evincible information [10]. Therefore, we excluded indeterminate values and preprocessed the data to avoid misinterpretation.

- The VAERS DATA dataset consists of features including ID, gender, age, vaccination date, and health history.
- The VAERS Symptoms dataset consists of adverse event symptoms by ID.
- The VAERS Vaccine dataset consists of ID, vaccine type, manufacturer, lot, number of doses administrated, route, site, and name.

### Data preprocessing

The VAERS datasets were merged and sorted by ID, with all duplicated ID rows being removed. We extracted only data whose vaccine type is COVID-19 vaccination. We used the data period from January 1 to August 31, 2021. We analyzed 396,443 cases of adverse events to the vaccine in our study. We divided the age variable into categories for simplicity. To ensure accurate analysis, all records with uncertain gender, age, vaccine manufacturer, and dose of vaccinations were removed.

In addition, the VAERS DATA contains information on what medications the vaccine recipients took; what allergies to medications, food, or other products they have; and what illnesses the vaccine recipients were suffering at the time of vaccination. Because these data values are in text format, the preprocessing is very important and complicated. First, data was converted to lowercase letters, then for tokenization, expressions like “and” and “;” were changed to “,”. Then we tokenized the data into units bounded by pairs of ‘,’. To eliminate any special characters in the data, we used regular expressions. Then, we compiled a list of words that meant “not applicable” such as “no”, “none”, “no reported”, “no known drug allergies” and “NKDA”, and converted these values to nulls. To remove unnecessary terminology, we used stopwords of the Natural Language Toolkit [24]. Furthermore, we removed inaccurate keywords in each feature. For example, the allergy feature contains symptoms like vomit and fever, and medication terms like disp and mg. In addition, most spelling errors and abbreviations were edited to unify word; for example, ‘penicillan’, ‘penecillin’, and ‘pennicillin’ were corrected to ‘penicillin’, and ‘htn’, ‘high blood pressure’, and ‘blood pressure high’ were unified to ‘hypertension’.

### Generating word embeddings using Word2Vec vectorizer

Word2Vec is a natural language processing technique (NLP) and word-embedding model developed by Google in 2013 [21]. Word2Vec uses the statistical properties of word relationships in certain contexts to extract semantic information by using vector spaces. Word2Vec is composed of two neural network-based language models: a continuous bag of words (CBOW) and a Skip-gram. The CBOW model is trained to predict the word in the center of a sliding window by using context words. Conversely, the Skip-gram model is trained to predict context words in the sliding window by using the word in the center [25, 26]. Skip-gram model produces better performances than CBOW model on semantic tasks [21].

Adverse events to the COVID-19 vaccine include a wide variety of symptoms, and the extensive range of variability of the dataset makes symptoms difficult to classify. To solve this challenge, symptom-related words were vectorized to create clusters grouped together with similar symptoms, then the characteristics of the clusters were analyzed. As Word2Vec yields good results for big data, it was used to generate large word vectors. There are hyperparameters in Word2Vec; Size means the size of the word vector; Window means the maximum distance between the current word and the predicted word; Minimum count means the minimum count threshold; iteration means the number of iterations (epochs) over the corpus; sg to 0 means the CBOW method, and 1 means the Skip-gram method. In our study, each hyperparameter was set to size to 500, window to 5, minimum count to 50, iteration to 100, and sg to 1, which is the appropriate architecture because symptom words are usually infrequent [27].

### Dimensionality reduction of word vectors via t-SNE

t-Distributed stochastic neighbor embedding (t-SNE) is an unsupervised learning model that is generally used for visualizing high-dimensional data [28]. It uses a Student-t distribution to compute the similarity between two points in low-dimensional space and transforms the *m*-dimensional data set $X=\{x_{1},x_{2},\ldots ,x_{t}\}\subset R^{m}$ to *n*-dimensional data $Y=\{y_{1},y_{2},\ldots ,y_{t}\}\subset R^{n}$, where *n*≪*m* and most commonly *n* = 2 or 3. We used the t-SNE algorithm to reduce the dimension to 2 to visualize this word vector.

### Clustering 2-D word vectors with the DBSCAN algorithm

DBSCAN is a non-parametric, clustering technique that considers density by hierarchical clustering and partitioning methods [22]. The basic assumption of DBSCAN is that a cluster is a high-density region in the data space [29].

We used the DBSCAN algorithm to ensure effective clustering. Two parameters of DBSCAN, eps and min samples, must be set to appropriate values, because they significantly affect the results. For this purpose, we used the KneeLocator package provided by Python to find knee points at specific minimum sample values then used them as the hyperparameter values of DBSCAN [30]. We set hyperparameters of the DBSCAN algorithm for eps to 0.686 and min samples to 5. Twenty-five clusters were created, but Cluster 0 was composed of residual data that were not otherwise classified, and was omitted. The characteristics of each of the other clusters were analyzed and visualized.

### Association rule analysis using the Apriori algorithm

The Apriori is a data mining algorithm that finds patterns indicating the degree to which specific items co-occur in one transaction; the algorithm is often used in association rule analysis [16]. Three scales are used to evaluate the associations as follows [31].

**Support**: The ratio of transactions that include items X and Y simultaneously among all items.


s(X⟹Y)=P(X,Y)ands(X)=P(X)

**Confidence**: when item X occurs, the probability that item Y will also occur.


c(X⟹Y)=P(Y|X)

**Lift**: A measure of whether a rule is caused by chance. If Lift is less than 1, X and Y have a negative relationship; if Lift is 1, X and Y are unrelated (that is, occur randomly relative to each other); and if Lift is greater than 1, X and Y have a positive relationship.


$$L(X⟹Y)=\frac{c(X⟹Y)}{P(Y)}=\frac{P(X,Y)}{\left(P(X)P(Y)\right)}$$

We performed association analysis using the Apriori algorithm to determine the regularity of symptoms that appear as adverse events to the COVID-19 vaccine. Minimum support is specified as a hyperparameter of the Apriori algorithm, which is the minimum support value of an itemset that can be a frequent itemset. However, it is varied that the range of the support count values of the symptom lists used as the input itemset. Therefore, if input consist of items that occur at low frequency, it can complicate that the algorithm discovers a meaningful association between them [32]. Consequently, to overcome this limitation of the Apriori algorithm, we created two transaction-type input datasets and set the minimum support for each transaction. First, Transaction-1 was constructed based on symptom lists in all clusters obtained from DBSCAN. Transaction-2 consisted of some clusters. We set hyperparameters of transactions with different minimum support thresholds as shown in Table 2. All results were displayed as network graphs using the Plotly package in Python.

**Table 2:** The hyperparameters of the apriori algorithm.

Hyperparameters	Transaction-1	Transaction-2
max_len	2	2
minimum support	0.01	0.001
minimum confidence	0.1	0.1
minimum lift	1	1

## Results

Table 3 summarizes the VAERS data collected from January 1 to August 31, 2021. Results indicated that there were approximately 2.5 times as likely to be female (72.0%) as to be male (28.0%) among people who experienced adverse events without normalizing for population size. Furthermore, of these people who experienced adverse events slightly more had used Moderna (51.3%) than Pfizer (41.6%), whereas very few had used Janssen (7.1%). Most vaccine doses were first and second. In addition, most of them were mainly in their 30s to 60s (67.4%); very few were under 10 or over 100 years old (0.1%).

**Table 3:** Summary of the VAERS dataset.

**Gender**	F	285,396
	M	111,047
**Vaccine Manufacturer**	Moderna	203,391
	Pfizer	165,061
	Janssen	27,991
**Vaccine dose**	1	255,897
	2	138,426
	3+	2,120
**Age**	0~9	265
	10~19	22,363
	20~29	43,526
	30~39	66,228
	40~49	65,949
	50~59	68,556
	60~69	66,407
	70~79	44,165
	80~89	15,434
	90~99	3,411
	100~	139

**Fig 2:**
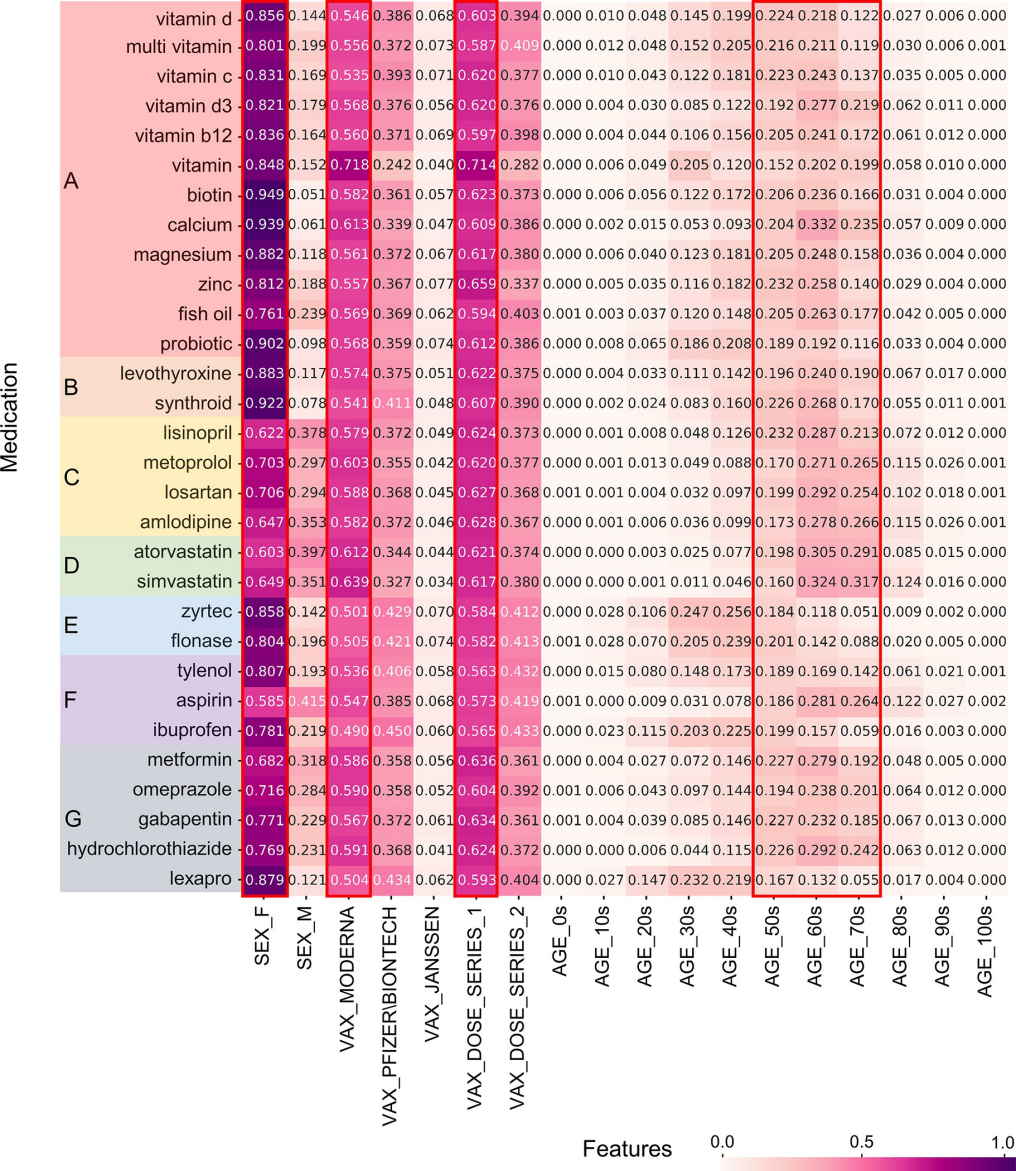
Summary of VAERS data concerning the top 30 medication keywords. We grouped medications with similar main active ingredients. (A) Dietary supplements; (B) Thyroid medications; (C) Hypertension medications; (D) High blood cholesterol medications; (E) Allergy medications; (F) Pain relievers; (G) Miscellaneous: Medications for Diabetes, Gastroesophageal reflux disease, Seizures, Diuretic, Depression.

**Fig 3:**
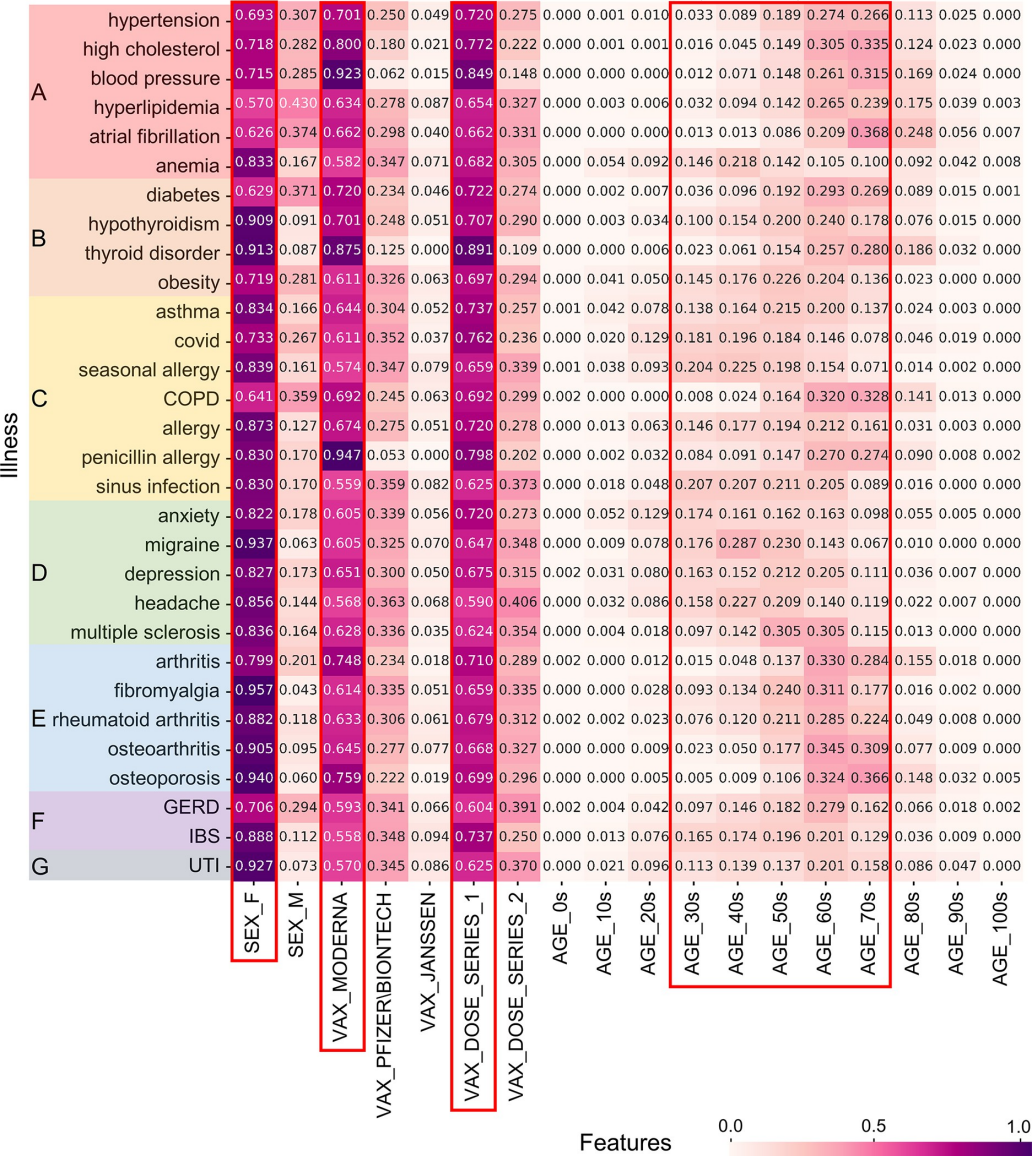
Summary of VAERS data concerning the top 30 illnesses keywords. We grouped illnesses by organ system. (A) Circulatory system; (B) Endocrine system; (C) Respiratory system; (D) Nervous System; (E) Musculoskeletal system; (F) Digestive system; (G) Urinary system. COPD, GERD, IBS, and UTI are abbreviations of Chronic Obstructive Pulmonary Disease, Gastroesophageal Reflux Disease, Irritable Bowel Syndrome, and Urinary Tract Infection, respectively.

**Fig 4:**
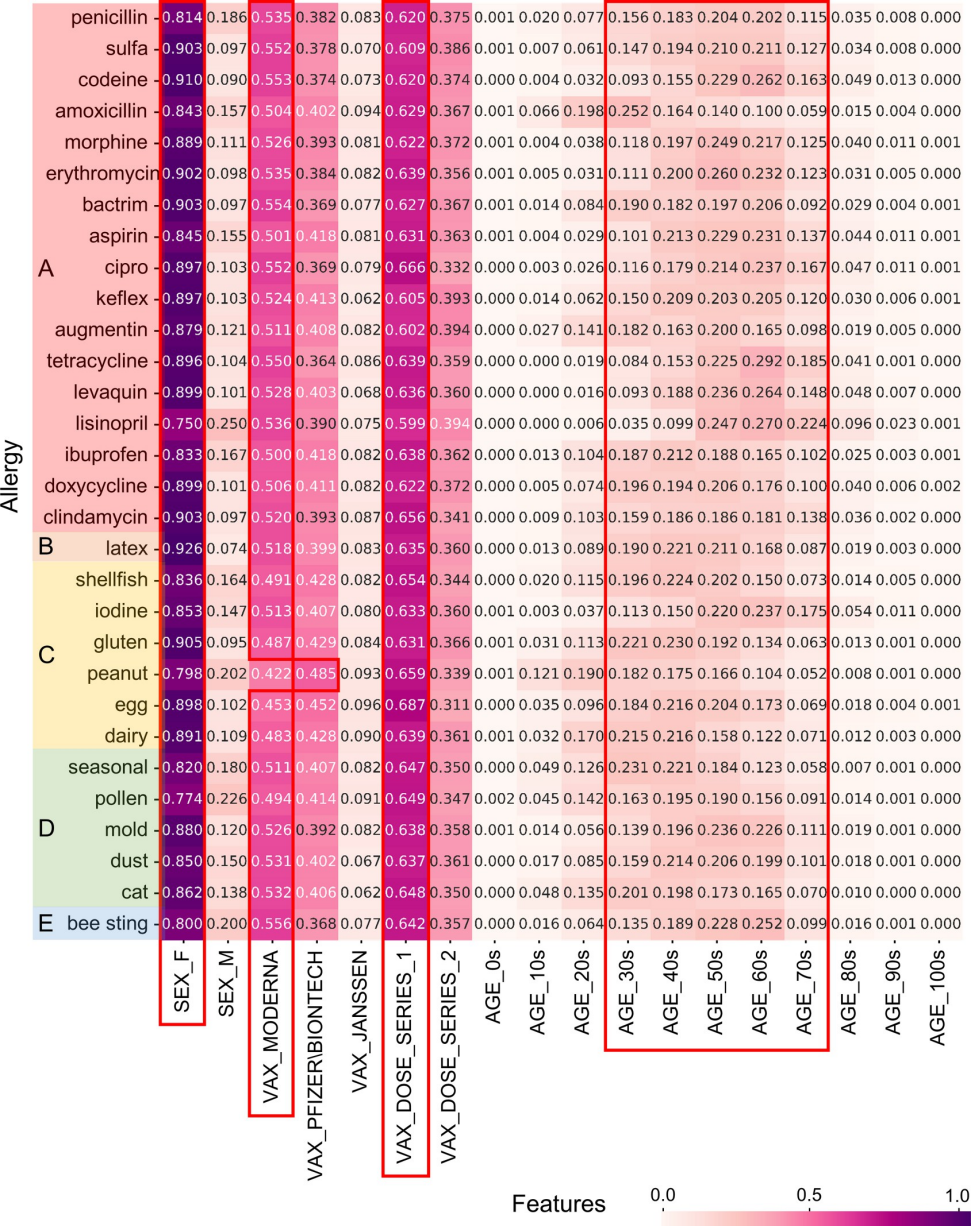
Summary of VAERS data concerning the top 30 allergies keywords. We grouped allergies by type of triggers. (A) Medications; (B) Materials; (C) Foods; (D) Airborne substances; (E) Insect venom.

Because the medication, illness, and allergy data of people who experienced adverse events were recorded as unstructured text from the narrative, a complex pre-processing was performed. Simple statistics for the top 30 keywords in each of the three health statuses were visualized using heatmaps, as shown in Figs 2–4. These figures represent the results of the normalized counting ratio of unique values in features including gender, vaccine manufacturers, vaccine does, and age for the top 30 keywords of medication, illness, and allergy, respectively.

Fig 2 lists statistics for the top 30 medications of what some people who experienced adverse events took at the time of vaccination. Vitamins ranked at the top, followed by thyroid medicines such as Levothyroxine and hypertension medicines including Lisinopril. For all medications, the results between genders and vaccine manufacturers groups were similar to those found in the overall dataset, whereas, in age groups, high rates of adverse events were associated with 50~70 years old for medications except for Zyrtec and Lexapro.

Fig 3 shows the top 30 illnesses of what some people who experienced adverse events suffered at the time of inoculation. The most common were Hypertension, Diabetes, Asthma, Covid, and Seasonal allergy. As in all other cases, the ratio was higher for women than for men, for Moderna than for Pfizer or Janssen, and for the first dose than for the second dose. Adverse events were also found to be high in middle-aged and older adults, mostly between their 50s and 70s; seasonal allergy, sinus infection, migraine, headache, and anemia were high in those between their 30s and 50s.

Fig 4 shows the top 30 allergies what some people who experienced adverse events had at the time of vaccination. Penicillin, Sulfa, Latex, Codeine, and Amoxicillin were ranked at the top tier. The results were similar to those described previously; however, patients who had a peanut allergy experienced the highest rates with the Pfizer vaccine. The age groups were evenly distributed from the 30s to the 70s.

To summarize the data statistics of these three health history attributes, the ratios were higher for women, Moderna, and the first dose vaccination than for others, and the number of patients who had adverse effects included a high number of those suffering from high blood pressure, thyroid, diabetes, and heart disease.

The VAERS Symptoms data identified 9,415 symptoms of adverse events to the COVID-19 vaccine; the most common were headache (97,219), pyrexia (81,095), fatigue (79,222), and chills (72,559). The diversity of the symptoms complicates the task of identifying and analyzing them. Therefore, we approached the VAERS dataset by grouping adverse event symptoms to analyze the vaccine recipients by symptoms. We vectorized the complete lists consisting of symptoms of adverse events using Word2Vec. As a result, Word2Vec generated 1,560 symptom vectors. We clustered these results by using DBSCAN after reducing the dimension of symptom embeddings through t-SNE. At this time, eps and min samples as the DBSCAN parameters were set to 0.686 and 5, respectively considering the result of KneeLocator.

**Fig 5:**
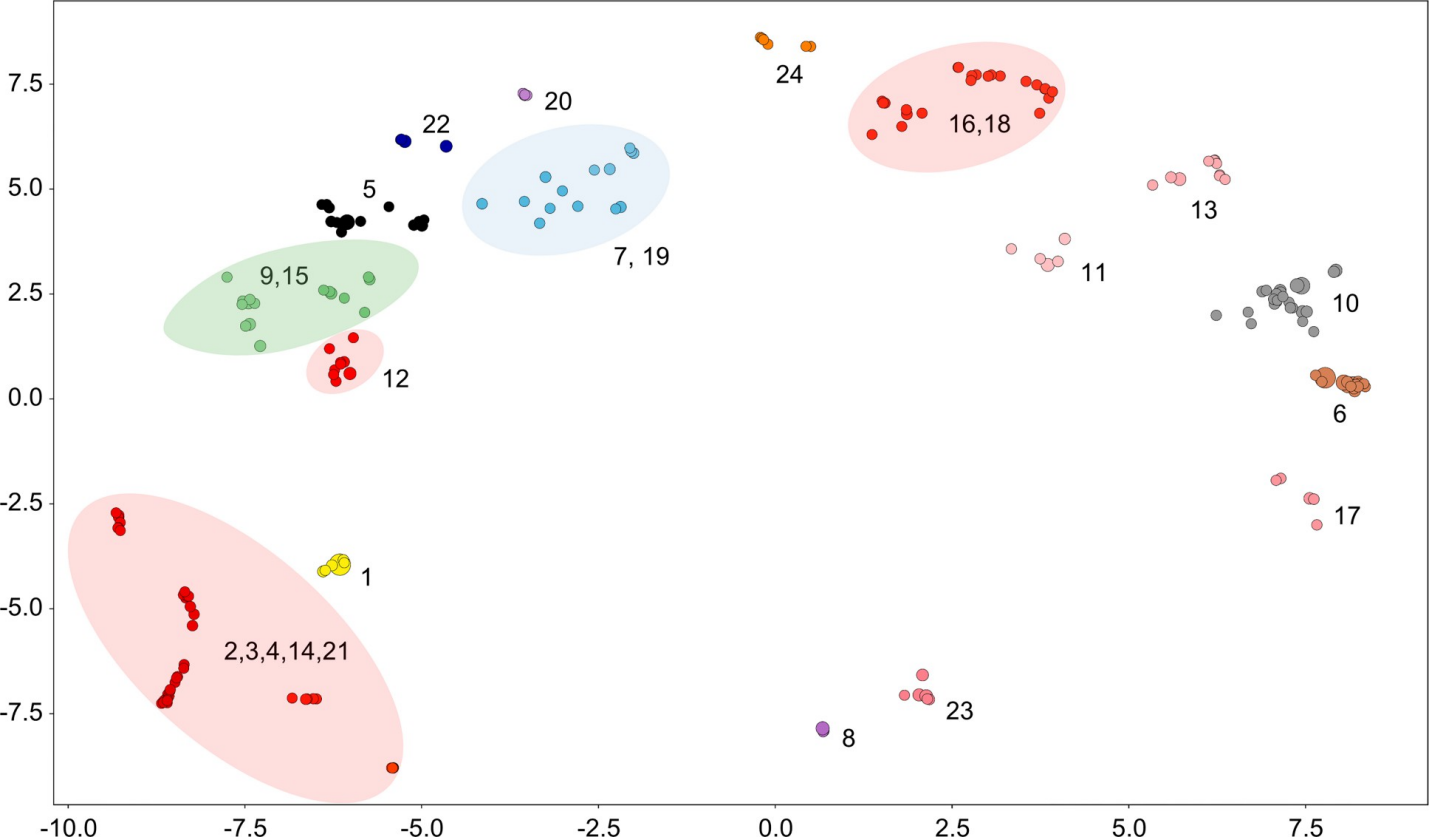
Results of clustering analysis of symptoms of adverse events to the COVID- 19 vaccine. Similar colors correspond to similar symptom groups.

The results of clustering symptoms are shown in Fig 5, and the five representative adverse event symptoms of each cluster are listed in Table 4. DBSCAN produced 25 clusters, but in this study, Cluster 0, which had no major characteristics, containing general symptoms like headache, pyrexia, and fatigue, was excluded. The remaining 24 clusters were composed of similar symptoms.

**Table 4:** Results of clustering analysis of symptoms of adverse events to COVID-19 vaccine.

Cluster	Symptoms
1	covid-19, covid-19 pneumonia, exposure to sars-cov-2, acute respiratory failure, asymptomatic covid-19
2, 3,4, 12, 14, 16, 18, 21	full blood count normal, platelet count normal, metabolic function test normal, hemoglobin normal, blood potassium normal
5	death, intensive care, cardiac arrest, endotracheal intubation, resuscitation
6	vaccination site pain, vaccination site erythema, vaccination site swelling, vaccination site pruritus, vaccination site warmth
7,19	oxygen saturation decreased, hypoxia, flatulence, lung opacity, flank pain
8	bell’s palsy, facial paralysis, facial paresis, facial asymmetry, corneal reflex decreased
9,15	troponin increased, myocarditis, troponin, pericarditis, troponin normal
10	product storage error, expired product administered, underdoes, product preparation issue, syringe issue
11	ultrasound scan, dysmenorrhea, ultrasound scan abnormal, ultrasound abdomen, surgery
13	exposure during pregnancy, abortion spontaneous, pregnancy, ultrasound fetal, glucose tolerance test
17	breast pain, breast swelling, breast tenderness, odynophagia, nipple pain
20	hallucination, visual, paranoia, auditory, psychotic disorder
22	pulmonary embolism, deep vein thrombosis, pulmonary thrombosis, pulmonary infarction, myocardial strain
23	heavy menstrual bleeding, menstruation irregular, menstrual disorder, polymenorrhea, oligomenorrhoea
24	urine leukocyte esterase, nitrite urine absent, specific gravity urine normal, urine ketone body absent, ph urine normal

Eight clusters (2, 3, 4, 12, 14, 16, 18, and 21; Fig 5, red) were related to blood tests. In Clusters 2, 3, 4, 14, and 21, hemoglobin normal and blood potassium normal were the main conditions. In Clusters 16 and 18, the main conditions were platelet count normal and mean cell volume normal, whereas the major condition of Cluster 12 was full blood count normal. Despite the differences, all clusters are related to blood test results.

Cluster 5 bundled serious outcomes such as death, intensive care, and cardiac arrest. Cluster 6 included symptoms related to vaccination site pain. Clusters 9 and 15 included heart-related symptoms such as myocarditis and pericarditis. Cluster 24 included symptoms related to urinalysis. Clusters 11, 13, 17, and 23 all exhibited symptoms that frequently occurred in women, including ultrasound scan, exposure during pregnancy, breast pain, and heavy menstrual bleeding. Cluster 10 included improper vaccination procedures such as product storage error and expired product administered.

We summarized the representative medications, illnesses, and allergies for each cluster. Medications seen in many clusters included levothyroxine (a treatment for the thyroid gland), lisinopril (a treatment for hypertension), atorvastatin (which lowers cholesterol), and vitamins. As shown in Fig 2, these medications were also among the top 30 represented in the overall dataset. Conversely, some medications that were not part of the top 30 (albuterol, furosemide, pantoprazole, prenatal vitamin, and spironolactone) appeared often in specific clusters. In particular, furosemide, a loop diuretic treatment for cardiac, renal, and hepatic failure, appeared frequently in Cluster 5.

Out of the top 30 illnesses represented by the dataset, those that appeared in the most clusters included hypertension, asthma, diabetes, and hypothyroidism. Conversely, illnesses outside of the top 30 that appeared in specific clusters included congestive heart failure, dementia, Downs syndrome, glaucoma, polycystic ovarian syndrome, ADHD, and pregnancy. In particular, congestive heart failure was seen most frequently in Cluster 5, and Downs syndrome was seen most frequently in Cluster 20. Furthermore, polycystic ovarian syndrome and pregnancy appeared mainly in the female disease cluster.

Finally, out of the top 30 allergies, penicillin, sulfa, amoxicillin, codeine, and latex appeared in the most clusters. Allergies outside of the top 30 that appeared in specific clusters included atorvastatin, statin, and tree nut allergies. In particular, statin allergies were counted the most frequently in Cluster 5. From the overall results, clusters associated with specific diseases exhibited different patterns commonly in medications, illnesses, and allergies.

Table 5 lists the number of vaccine adverse events varied by gender and vaccine manufacturer. For gender, the ratio of females was high in most clusters except 5, 9, and 15. For vaccine manufacturers, Moderna and Pfizer had a similar ratio; this result differed from the initial result due to the exclusion of Cluster 0. Table 5 also can be interpreted by considering average values. Clusters 6, 11, 13, 17, and 23, which all exhibited higher values than the average female ratio, were symptom clusters related to vaccine site pain and gynecologic diseases. The Moderna ratios in Clusters 6, 13, and 20 were higher than its average ratio. For Pfizer and Janssen, many Clusters showed a higher rate than their average respectively. Thus, the ratio in each cluster exhibited a slightly different ratio from the overall ratio.

**Table 5:** Summary of the number of vaccine adverse events and the proportions by gender and vaccine manufacture in each cluster.

Cluster	Number	Female	Male	Moderna	Pfizer	Janssen
1	8,911	0.593	0.407	0.368	0.553	0.079
2, 3,4, 12, 14, 16, 18, 21	3,700	0.635	0.365	0.403	0.481	0.116
5	5,053	0.443	0.557	0.482	0.430	0.088
6	16,121	0.822	0.178	0.728	0.269	0.003
7, 19	3,181	0.602	0.398	0.461	0.460	0.079
8	3,199	0.591	0.409	0.423	0.503	0.074
9,15	4,238	0.450	0.550	0.366	0.568	0.066
10	9,980	0.545	0.455	0.384	0.563	0.053
11	3,636	0.795	0.205	0.417	0.463	0.120
13	2,375	0.997	0.003	0.563	0.385	0.052
17	1,616	0.936	0.064	0.421	0.527	0.052
20	776	0.651	0.349	0.526	0.376	0.098
22	2,524	0.532	0.468	0.412	0.404	0.184
23	3,813	0.998	0.002	0.342	0.567	0.091
24	121	0.669	0.331	0.471	0.438	0.091

Next, the adverse events in each cluster were compared by age group. These events were mainly distributed in the 30s to 60s, Clusters 1, 8, 11, 17, 20, and 22 displayed a similar trend. However, Clusters 9, 15, 13, and 23 were more frequently represented by the younger group than the older group, whereas Clusters 5, 6, 7, and 19 mostly consisted of the elderly. Pregnancy-related Cluster 13 and menstruation-related Cluster 23 usually included a specific group (female and twenty to forty), however 9.4% of Cluster 23 were women over 50.

Association analysis was conducted to identify symptoms of the various COVID-19 vaccine adverse events that occur together. The Apriori algorithm was used to perform two association analyses with different input datasets. Because the distribution of symptoms was unbalanced (e.g., headache appeared 76,900 times, whereas bell’s palsy appeared 2,044 times), we built two transactions with different compositions of the items. Transaction-1 included all symptoms observed in DBSCAN clustering, and Transaction-2 excluded the categories of product errors, medical test results, and all of cluster 0, which contained symptoms with a large number of appearances in common. In addition, we set the hyperparameters of each transaction differently and set the minimum support value smaller for Transaction-2, which consisted of symptoms with a low frequency of appearance, than for Transaction-1.

**Fig 6:**
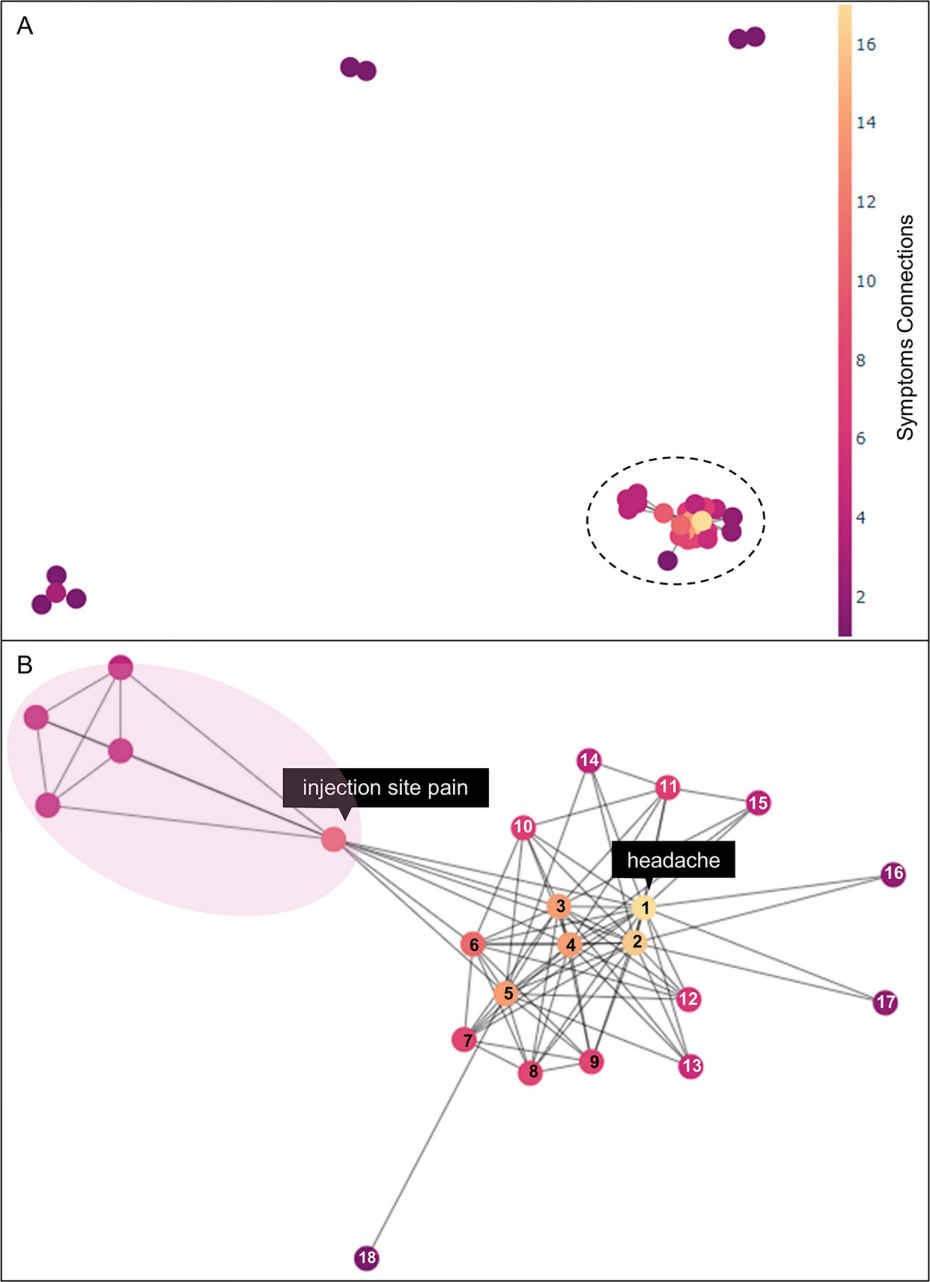
The network is the result of the association analysis of Transaction-1. (A) Overall graph of Transaction-1. (B) Expansion of the graph in (A). On the left side of (B), the red areas are connected with four symptoms associated with injection site pain; injection site erythema, injection site pruritus, injection site swelling, and injection site warmth. In the right side of (B), several symptoms are linked with a headache, and the following symptoms are indicated in the order of highest number of connections; headache, fatigue, nausea, chills, pyrexia, pain, pain in extremity, myalgia, arthralgia, asthenia, dizziness, vomiting, diarrhoea, dyspnoea, hyperhidrosis, feeling abnormal, malaise, and body temperature.

**Fig 7:**
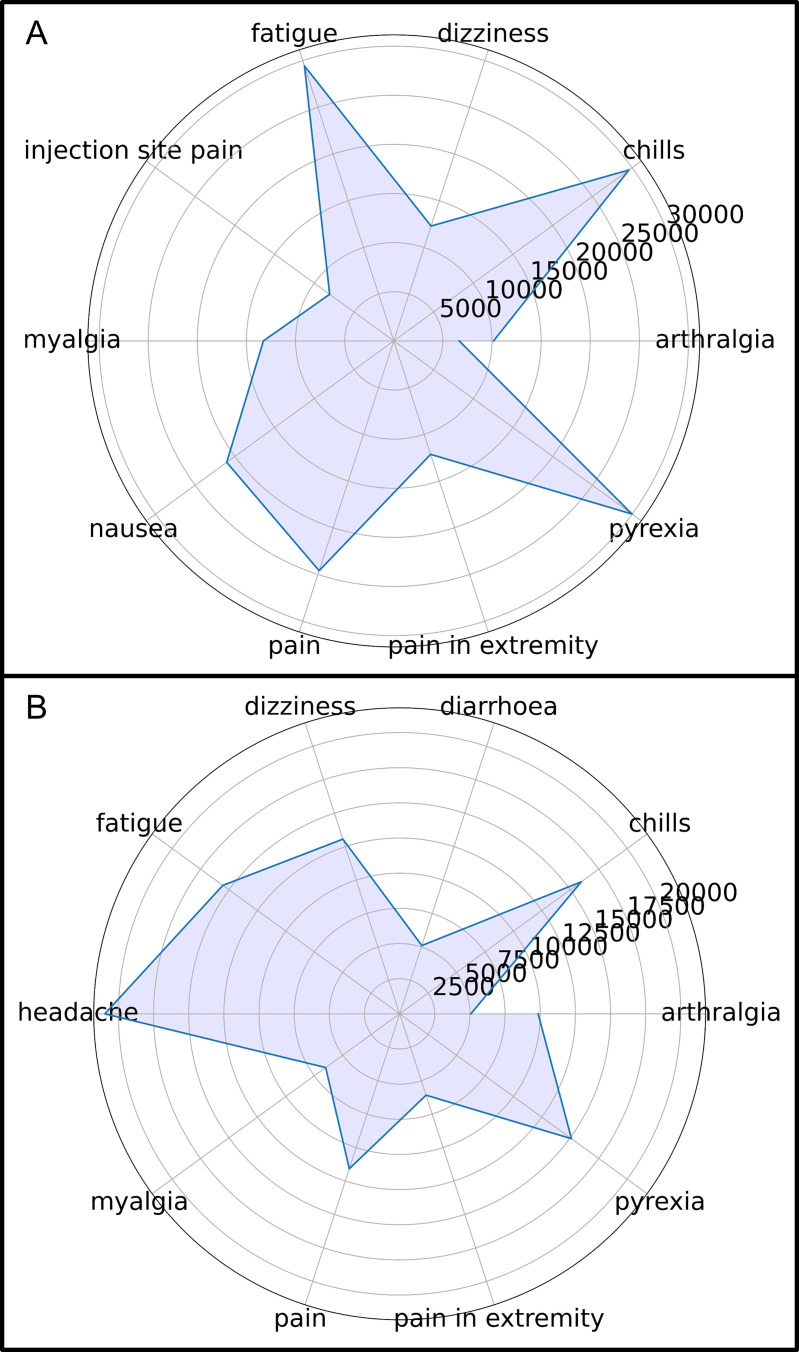
Polar plot visualizing statistical analysis results based on association analysis results.

First, we conducted the association analysis of Transaction-1 for all symptoms as shown in Fig 6. Fig 6A displays a graph of all the rules, and Fig 6B shows an enlargement of the most connected rules of Fig 6A. Fig 6B displays an association map between the 23 most common adverse event symptoms. Among them, the symptom that had the highest comorbidity with other symptoms was headache, which was linked to 17 symptoms. The highest support value was 0.087, which was represented by the {chills ↔ pyrexia} rule. The highest confidence value was 0.858, represented by the {body temperature → pyrexia} rule. As shown in Fig 7, symptom association patterns were statistically calculated using all symptom data and visualized as a polar plot. Fig 7A shows that the most common symptoms accompanying headache were pyrexia, chills, and fatigue. Rules with antecedent to headache also showed high support and confidence values in a similar order. Fig 7B shows that the symptoms most associated with nausea were headache, chills, and fatigue. Support and confidence values of nausea for those were also high.

**Fig 8:**
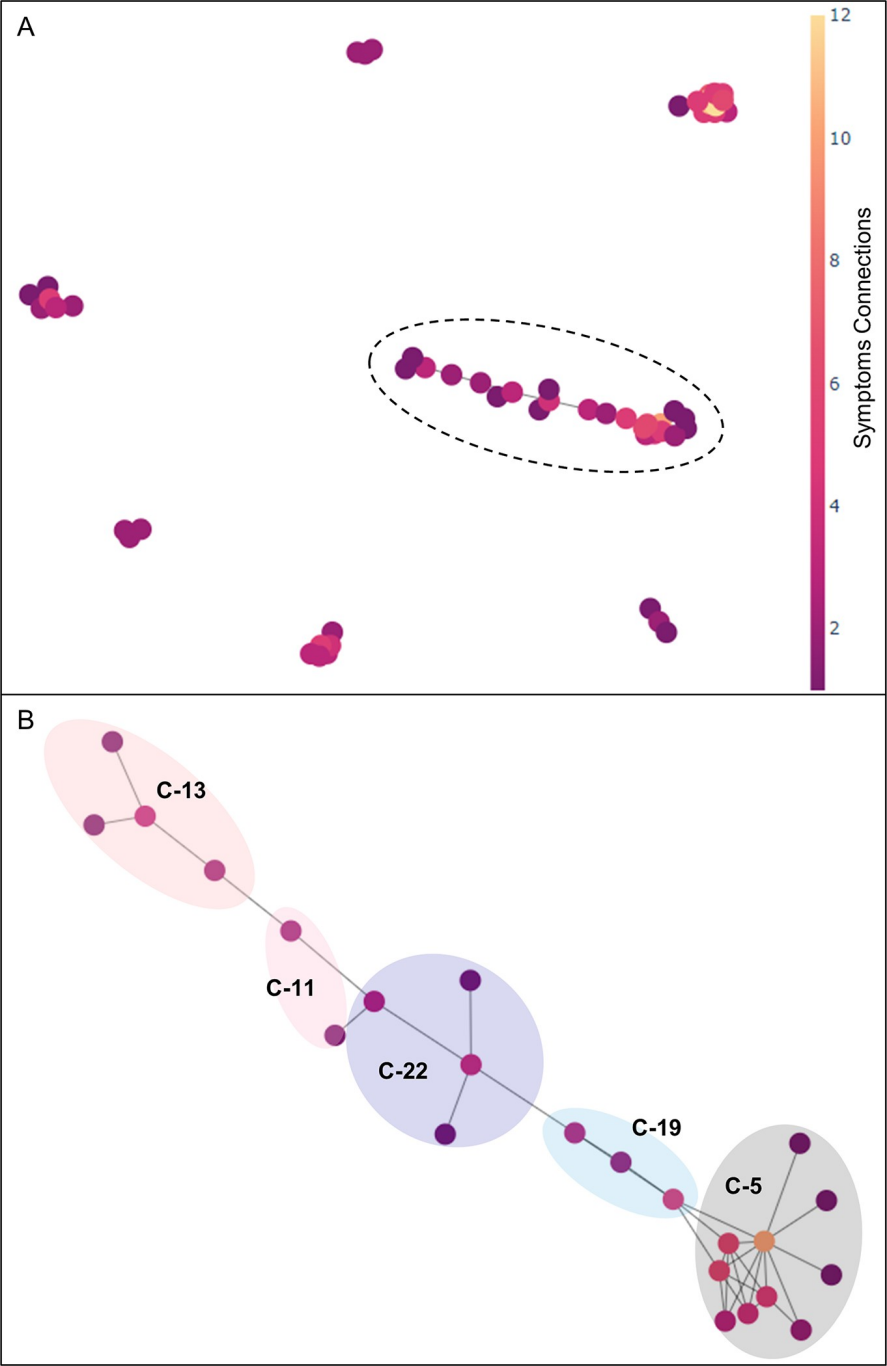
The network of association rules of Transaction-2. (A) Overall graph of Transaction-2. (B) Enlargement of the graph in (A). Clusters are distinguished by color. Symptoms corresponding to each cluster are as follows. Cluster 13: exposure during pregnancy, abortion spontaneous, ultrasound antenatal screen abnormal, and ultrasound fetal; Cluster 11: ultrasound scan and ultrasound scan abnormal; Cluster 22: pulmonary embolism, deep vein thrombosis, myocardial strain, and pulmonary infarction; Cluster 19: hypoxia, lung opacity, and lung infiltration; Cluster 5: death, endotracheal intubation, intensive care, resuscitation, cardiac arrest, mechanical ventilation, pulse absent, autopsy, general physical health deterioration, and pulseless electrical activity.

Transaction-2 was constructed considering 13 clusters: 5, 6, 7, 8, 9, 11, 13, 15, 17, 19, 20, 22, and 23. Table 6 displays examples of Transaction-2. The results of the association analysis on Transaction-2 are shown in Fig 8. Fig 8A shows a graph of all the rules. In Fig 8A, the area with the most symptoms is shown in Fig 8B. Death was associated with the most symptoms, connected to five symptom clusters in Fig 8B. The highest support value was 0.046, corresponding to the {vaccination site pruritus ↔ vaccination site erythema} rule. The highest confidence value was 1, corresponding to the {visual → hallucination} rule and {auditory → hallucination} rule.

**Table 6:** Transaction-2 based on the results of DBSCAN clustering.

dbscan	Symptom sets
5	**death**, pulmonary embolism, troponin increase
6	**vaccination site pain**, oxygen saturation decreased, flank pain
7,19	**oxygen saturation decreased**, pulse absent, hypoxia
8	**bell’s palsy**, polymenorrhoea, hallucination, facial paralysis
9,15	**troponin increased**, myocarditis, pericarditis, intensive care
11	**ultrasound scan**, abortion spontaneous, exposure during pregnancy
13	**exposure during pregnancy**, fetal heart rate abnormal, ultrasound antenatal screen
17	**breast pain**, vaccination site pain, breast tenderness, breast swelling
20	**hallucination**, endotracheal intubation
22	**pulmonary embolism**, lung opacity, deep vein thrombosis
23	**heavy menstrual bleeding**, vaccination site pain, menstruation irregular

## Conclusion

The VAERS data is a more useful dataset in that vaccination is an important approach to preventing ever-increasing infectious diseases along with non-pharmacological interventions. Consequently, for any analysis using the VAERS dataset, competent data preprocessing is a very important first step, and our results demonstrated proper conduct.

According to the VAERS symptoms data, the most common symptoms of COVID-19 vaccine adverse events are headache, pyrexia, fatigue, chills, and pain. We performed symptoms vectorization using Word2Vec. To identify various other symptoms, we reduced the dimension of embedding vectors and performed clustering experiments on the vectors by using the DBSCAN algorithm. The process identified 25 symptom clusters, but cluster 0 was a generic group, and was excluded from further analysis. Various symptoms such as pain at the injection site, gynecologic disease, lung and heart disease, and death were clustered.

Furthermore, we extrapolated that the vaccine adverse events of COVID-19 were evidently different from the same respiratory virus, Influenza vaccine, as obtained from the VAERS data. According to the data, the main symptoms of the Influenza vaccine adverse events are injection site pain, pain, and pyrexia [10]. Also, the symptoms were less diverse than for the COVID-19 vaccine.

We summarized various symptom clusters of adverse events to the COVID-19 vaccine. Moreover, our study concludes that symptom clusters have different characteristics according to gender, vaccine manufacturer, and age. For example, Clusters 9 and 15 included a higher-than-average ratio of young males who received the Pfizer vaccine. Consequently, it is necessary to be aware that symptoms may appear depending on the underlying diseases (medications, illnesses, and allergies) of the person receiving the vaccine. Notably, Cluster 5 included recipients who have taken furosemide, or who have congestive heart failure, even though the percentages were small.

In addition, the results of the association analysis indicated that headache occurred with mild symptoms including fatigue, chills, pyrexia, and nausea. Moreover, the findings suggest that death is significantly related to Cluster 19. We expect that this study can help to anticipate adverse events that a vaccinated person might experience, and to prepare for them.

Our study presents overall symptoms that can appear as adverse reactions following COVID-19 vaccination, and demonstrates the characteristics of groups who have specific symptoms. However, our study has the limitation that the VAERS data only includes people who had adverse reactions among vaccinated people. In other words, it cannot determine whether adverse events will occur even if vaccine recipients have similar characteristics to the patient in this data. In addition, since the human body is complicated system, causal relationships cannot be confirmed unconditionally.

Therefore, our results provide overall factual information and trends that can be presented to people who are deciding whether to vaccinate, considering the experiences of previous recipients, and can facilitate preparation for vaccination. In addition, we contribute to developing shreds of evidence to verify the authenticity of uncertain information related to adverse events of COVID-19 vaccines.

In further studies, we expect to access additional data sources such as VAERS data from other countries. We will also continue to study the predictability of adverse events by using machine-learning techniques with additional datasets.
